# Overexpression of pig selenoprotein S blocks OTA-induced promotion of PCV2 replication by inhibiting oxidative stress and p38 phosphorylation in PK15 cells

**DOI:** 10.18632/oncotarget.7814

**Published:** 2016-03-01

**Authors:** Fang Gan, Zhihua Hu, Yu Huang, Hongxia Xue, Da Huang, Gang Qian, Junfa Hu, Xingxiang Chen, Tian Wang, Kehe Huang

**Affiliations:** ^1^ College of Veterinary Medicine, Nanjing Agricultural University, Nanjing 210095, Jiangsu Province, China; ^2^ Institute of Nutritional and Metabolic Disorders in Domestic Animals and Fowls, Nanjing Agricultural University, Nanjing 210095, Jiangsu Province, China; ^3^ College of Animal Science and Technology, Nanjing Agricultural University, Nanjing 210095, Jiangsu Province, China

**Keywords:** overexpression of selenoprotein S, ochratoxin A, porcine circovirus type 2, oxidative stress, p38 signaling pathway

## Abstract

Porcine circovirus type 2 (PCV2) is the primary cause of porcine circovirus disease, and ochratoxin A (OTA)-induced oxidative stress promotes PCV2 replication. In humans, selenoprotein S (SelS) has antioxidant ability, but it is unclear whether SelS affects viral infection. Here, we stably transfected PK15 cells with pig pCDNA3.1-SelS to overexpress SelS. Selenium (Se) at 2 or 4 μM and SelS overexpression blocked the OTA-induced increases of PCV2 DNA copy number and infected cell numbers. SelS overexpression also increased glutathione (GSH), NF-E2-related factor 2 (Nrf2) mRNA, and γ-glutamyl-cysteine synthetase mRNA levels; decreased reactive oxygen species (ROS) levels; and inhibited p38 phosphorylation in PCV2-infected PK15 cells, regardless of OTA treatment. Buthionine sulfoximine reversed all of the above SelS-induced changes. siRNA-mediated SelS knockdown decreased Nrf2 mRNA and GSH levels, increased ROS levels, and promoted PCV2 replication in OTA-treated PK15 cells. These data indicate that pig SelS blocks OTA-induced promotion of PCV2 replication by inhibiting the oxidative stress and p38 phosphorylation in PK15 cells.

## INTRODUCTION

Porcine circovirus type 2 (PCV2), a single-stranded DNA virus, is the primary causative agent of several syndromes collectively known as porcine circovirus disease (PCVD) [1]. This cluster of diseases, which includes postweaning multisystemic wasting syndrome, porcine respiratory disease complex, and porcine dermatitis and nephropathy syndrome, results in losses of up to 20 dollars per pig in the United States [2]. However, not all pigs infected with PCV2 develop PCVD, and the severity of the disease differs among pig farms. It has been reported that PCVD occurrence is associated with animal management practices, the presence of concurrent viral infections, nutrition, and other factors [3]. Our previous work indicated that oxidative stress enhanced PCV2 replication *in vitro* [4], and ochratoxin A (OTA) promoted PCV2 replication both *in vitro* and *in vivo* [5], partly explaining differences in morbidity and severity of PCVD in PCV2-infected pigs.

Selenium (Se) is an essential trace element in humans and animals [6-8] and has antioxidant functions [9]. Se deficiency increases carcinogenesis and hepatitis C virus, influenza virus, and HIV infections in humans and animals [10-12]. Meanwhile, Se supplementation inhibits viral infections, including PCV2 [13-16]. Se exerts its biological functions through selenoproteins, such as glutathione peroxidase (GPx), thioredoxin reductases (TRs), and endoplasmic-reticulum selenoproteins [17], all of which participate in antioxidant defense and redox signaling [18]. We previously reported that GPx1 knockdown promoted PCV2 replication and reversed the ability of Se to block hydrogen peroxide (H_2_O_2_) -induced PCV2 replication [15]. However, further work is needed to determine the roles of other selenoproteins in PCV2 replication.

Selenoproteins are a small but vital family of proteins that contain selenocysteine (Sec) as their 21st amino acid residue [19]. The most remarkable trait of selenoprotein biosynthesis is the cotranslational insertion of Sec by the recoding of a naturally occurring UGA stop codon [20, 21]. The Sec Insertion Sequence (SECIS) element located in the 3′-UTRs of all selenoprotein mRNAs is required for incorporation of Sec into nascent selenoprotein polypeptides in response to the UGA codon [22, 23]. Cloning selenoproteins is difficult due to this characteristic. Although overexpression of some selenoproteins has been reported in humans and mice [24, 25], there are few reports of selenoprotein overexpression in pigs.

Selenoprotein S (SelS), an important selenoprotein, is expressed in a pancreatic β cell line, human endothelial cells (ECs), and porcine liver, kidney, and muscle [26-29]. High SelS levels protected pancreatic β cells and human ECs from H_2_O_2_-induced oxidative injury [27, 30]. Additionally, SelS knockdown increased H_2_O_2_-induced oxidative injury and decreased cell viability in human ECs [30]. High SelS levels also inhibited, and SelS silencing increased, H_2_O_2_-induced oxidative stress in vascular smooth muscle cells [31]. These reports indicate that SelS has antioxidation in humans. However, SelS overexpression and the relationship between SelS and virual infection in pigs are unknown.

Here, we constructed PK15 cell lines that overexpress SelS to investigate whether, and by what underlying mechanisms, SelS affects the OTA-induced promotion of PCV2 replication. We hypothesized that: (i) pig SelS has antioxidant ability, (ii) SelS overexpression could block OTA-induced promotion of PCV2 replication in PK15 cells, (iii) SelS is important for the ability of Se to block this type of PCV2 replication, and (iv) the blocking effects of SelS may be due to its actions on the oxidative stress-mediated p38 and ERK1/2 MAPK signaling pathways.

## RESULTS

### Construction of the SelS overexpression plasmid, pc-SelS

**Figure 1:**
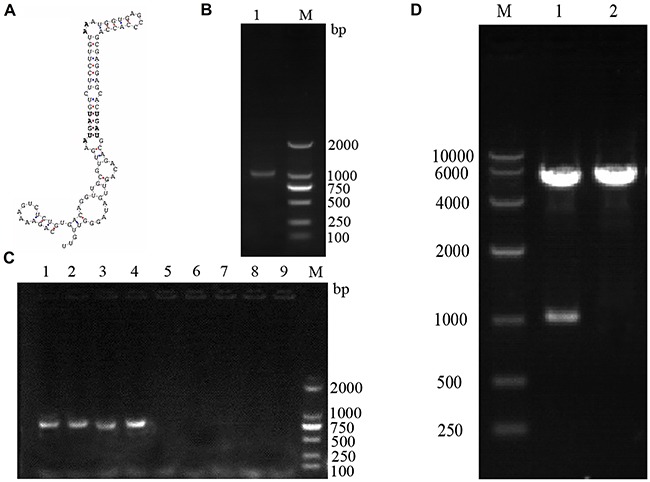
Construction of the SelS overexpression plasmid, pc-SelS SECISearch software was used to identify the SECIS sequence of porcine SelS **A.** A single target SelS gene, 1029 bp in length, was identified using PCR and electrophoresis **B.** pcDNA3.1-SelS was verified using colony PCR **C.** and restriction endonuclease digestion **D.**

As shown in Figure 1, the SECIS sequence in the pig SelS 3′-UTR was identified using SECISearch software (Figure 1A). Total RNA was extracted from pig kidney tissue and reversed transcribed into cDNA, which was then amplified with PCR using a SelS primer; electrophoresis showed that the product was a single target SelS gene 1029bp in length (Figure 1B). The eukaryotic SelS overexpression plasmid, pc-SelS, was constructed using a pcDNA3.1 vector and was verified by colony PCR (Figure 1C) and restriction endonuclease digestion and DNA sequencing (Figure 1D).

### Construction PK 15 cell lines overexpressing SelS

**Figure 2:**
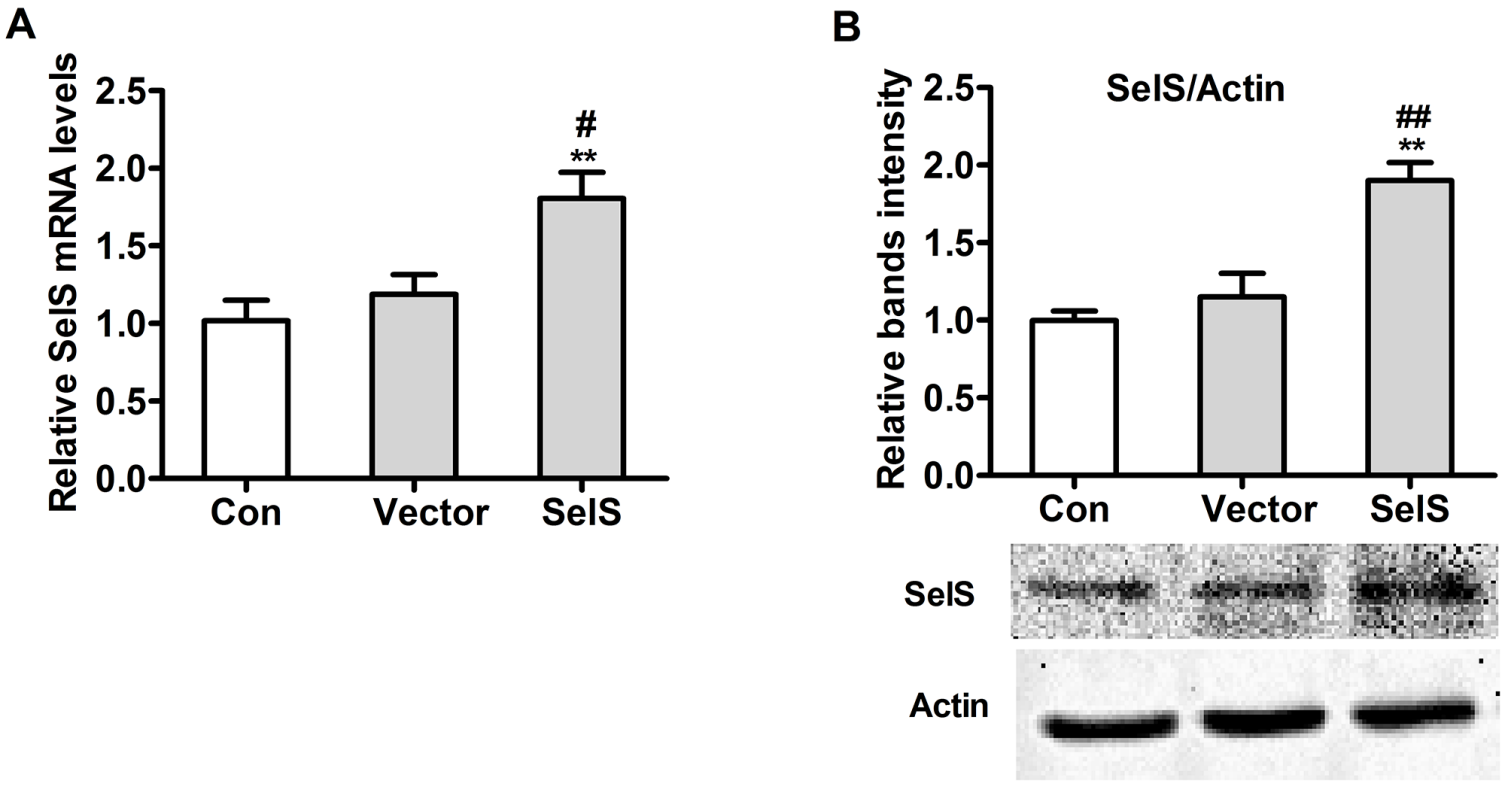
Expression of the pc-SelS in PK 15 cells SelS mRNA **A.** and protein **B.** levels after transfecting pc-SelS into PK15 cells were determined using real-time PCR and western blotting as described in Materials and Methods. Data are presented as means ± SE of three independent experiments. *P < 0.05 and **P < 0.01 vs. control. ^#^*P* < 0.05 and ^##^*P* < 0.01 vs. vector control.

The pig pc-SelS plasmid was stably transfected into PK15 cells and resulted in the overexpression of SelS. As shown in Figure 2, transfection of the SelS plasmid into PK15 cells increased SelS mRNA (Figure 2A) and protein (Figure 2B) levels as compared to control and empty vector-transfected cells.

### SelS overexpression increased antioxidant ability in PK15 cells

**Figure 3:**
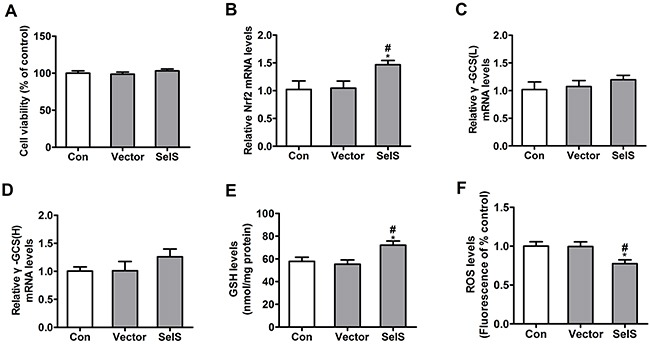
SelS overexpression increased antioxidant ability in PK15 cells SelS-overexpressing PK15 cells were incubated for 72 h in DMEM. The cell viability **A.** Nrf2 mRNA levels **B.** γ-GCS mRNA levels **C, D.** GSH levels **E.** and ROS levels **F.** were assayed as described in the Materials and Methods. Data are presented as means ± SE of three independent experiments. *P < 0.05 and **P < 0.01 vs. control. *^#^P* < 0.05 and ^##^P < 0.01 vs. vector control.

To determine whether SelS overexpression increases antioxidant ability, Nrf2 mRNA, γ-GCS mRNA, GSH, and ROS levels were measured in PK15 cells. Cells were seeded in 96- and 6-well plates at densities of 4 × 10^3^ and 2 × 10^5^ cells/well, respectively, and were cultured for 72 h. As shown in Figure 3, viability was similar in PK15 cells, vector-PK15 cells, and SelS-PK15 cells (Figure 3A). SelS overexpression did not affect γ-GCS mRNA levels (Figure 3C, 3D), but increased Nrf2 mRNA (Figure 3B) and GSH levels (Figure 3E), and decreased ROS levels (Figure 3F) compared to the control and empty vector groups. These results suggest that SelS overexpression increases antioxidant ability in PK15 cells.

### Se supplementation and SelS overexpression blocked OTA-promoted PCV2 replication

**Figure 4:**
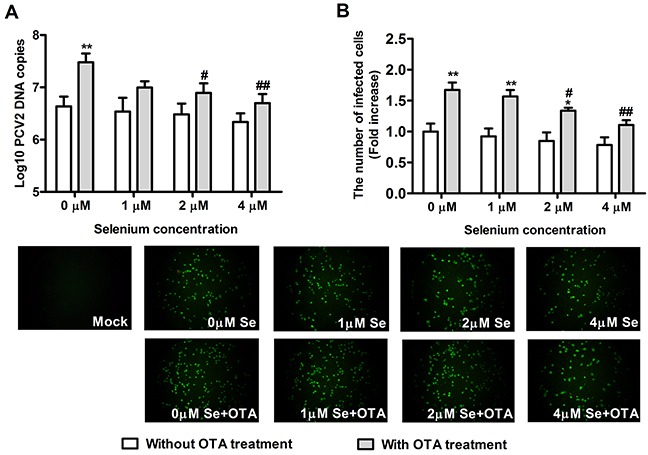
Se supplementation blocked OTA-promoted PCV2 replication PK15 cells were cultured for 12 h with 0, 1, 2 or 4 μM Se and then incubated for an additional 60 h with PCV2 in the presence or absence of 0.05 μg/ml OTA. Cells were assayed for PCV2 DNA copies **A.** using real-time PCR and the number of infected cells **B.** using IFA. Data are presented as means ± SE of three independent experiments. *P < 0.05 and **P < 0.01 vs. control (without OTA or Se). Within the OTA treatment groups, *^#^P* < 0.05 and ^##^*P* < 0.01 vs. control cells without Se.

**Figure 5:**
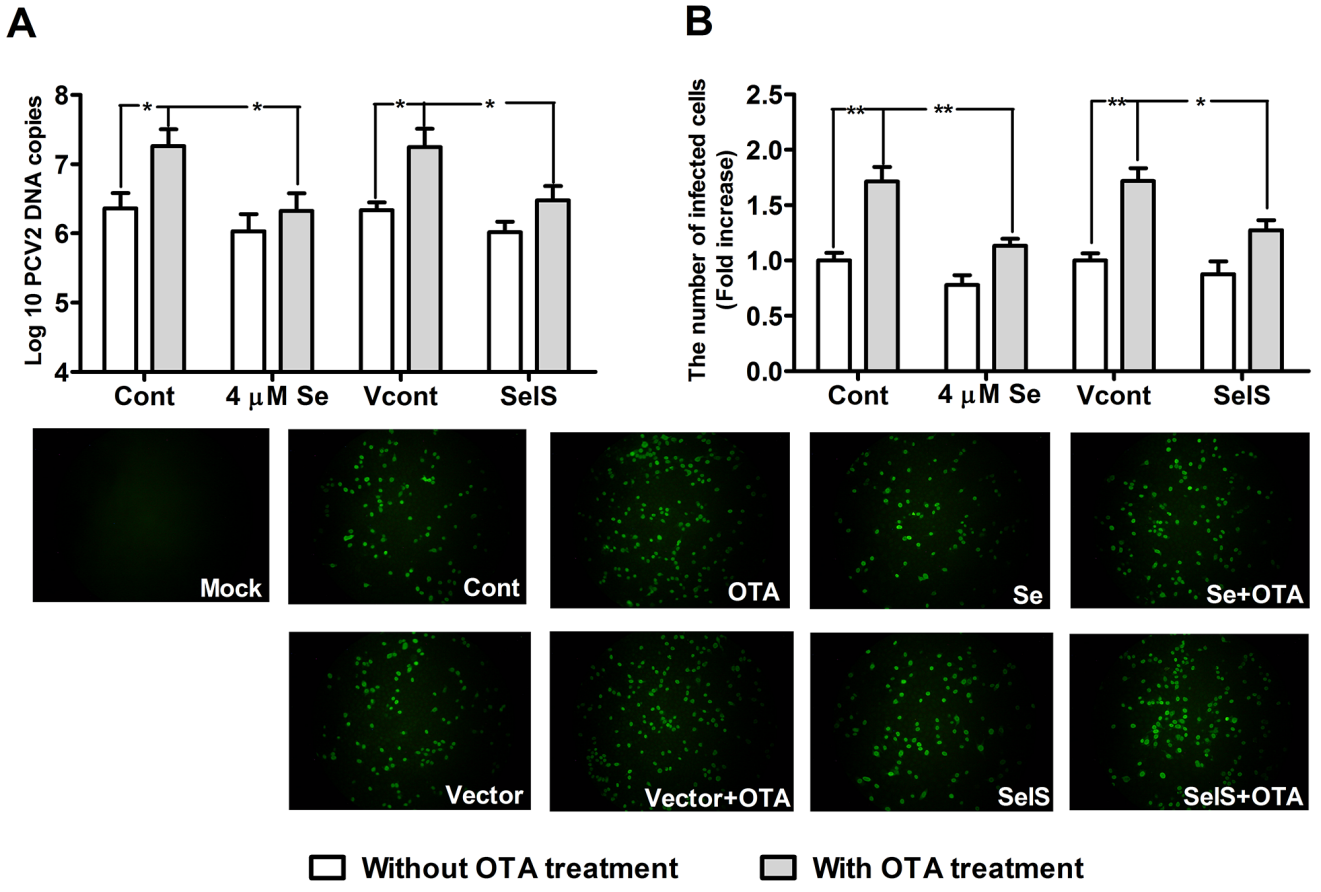
SelS overexpression blocked OTA-promoted PCV2 replication PK15 cells were cultured for 12h with or without 4 μM Se and then incubated for an additional 60 h with PCV2 in the presence or absence of 0.05 μg/ml OTA. Cells were assayed for PCV2 viral DNA copies **A.** using real-time PCR and the number of infected cells **B.** using IFA. Data are presented as means ± SE of three independent experiments. *P < 0.05 and **P < 0.01 vs. the respective Cont and Vcont groups.

To determine whether Se supplementation and SelS overexpression inhibit PCV2 replication in PK15 cells, we measured PCV2 DNA copy and the infected cell numbers. Cells at densities of 5 × 10^4^/well in 12-well plates and 5 × 10^3^/well in 96-well plates were cultured with or without Se for 12h, and were then incubated with PCV2 in the presence or absence of 0.05 μg/ml OTA for an additional 60 h. As shown in Figure 4 and Figure 5, in cells without OTA treatment, Se at 1, 2, or 4 μM and SelS overexpression tended to decrease PCV2 DNA copy and infected cell numbers compared to the respective control group (*P* > 0.05), but this difference was not significant. In addition, 0.05 μg/ml OTA significantly increased PCV2 DNA copy and infected cell numbers. Se at 2 or 4 μM blocked OTA-induced PCV2 replication promotion compared to the control group (Figure 4A, 4B) (*P* < 0.05). SelS overexpression also blocked OTA-induced PCV2 replication promotion compared to the vector control group (Figure 5A, 5B) (*P* < 0.05). Furthermore, Se blocked the PCV2 replication to a greater degree than SelS overexpression. These results indicate that Se and SelS overexpression both block OTA-induced PCV2 replication promotion, suggesting that SelS may be partly responsible for the blocking effect of Se.

### Effects of OTA treatment or/and PCV2 infection on oxidative stress

**Figure 6:**
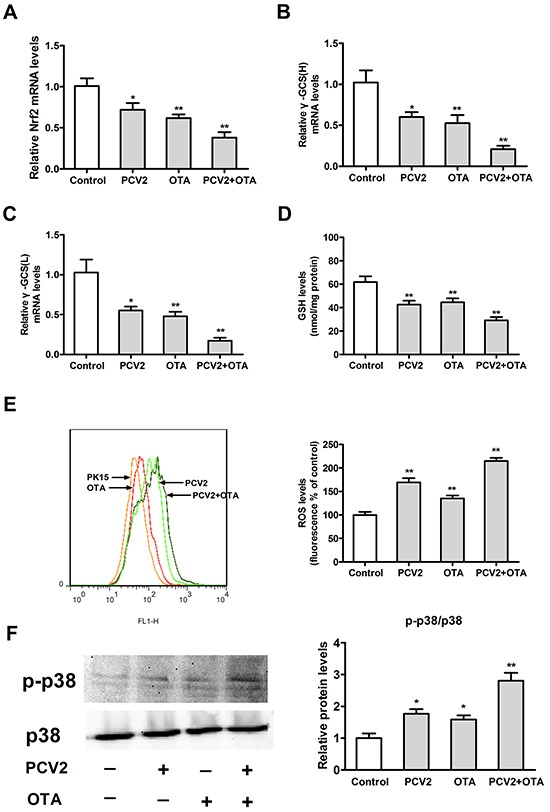
Effects of OTA treatment and/or PCV2 infection on oxidative stress Vector-PK15 cells were incubated for 24 h with or without PCV2 and then for 48 h in the presence or absence of 0.05 μg/ml OTA. Cells were harvested after an additional 48 h in the presence of OTA. Levels of Nrf2 **A.** and γ-GCS **B, C.** mRNA, GSH **D.** ROS **E.** and p38 phosphorylation **F.** were assayed as described in the Materials and Methods. Data are presented as means ± SE of three independent experiments. *p < 0.05 and **p < 0.01 vs. control.

To measure the oxidative stress induced by OTA treatment or/and PCV2 infection, we examined levels of ROS, GSH, and p38 phosphorylation. Vector-PK15 cells at a density of 2 × 10^5^/well in 6-well plates were inoculated with PCV2 at an MOI of 1 for 72 h or/and OTA at a concentration of 0.05 μg/ml for 48 h. As shown in Figure 6, PCV2 infection or OTA treatment significantly decreased Nrf2 mRNA (Figure 6A), γ-GCS mRNA (Figure 6B, 6C), and GSH levels (Figure 6D), and increased ROS levels (Figure 6E) and p38 phosphorylation (Figure 6F). In addition, OTA treatment plus PCV2 infection enhanced all of these changes. These results suggest that OTA treatment or/and PCV2 infection induces oxidative stress.

### SelS overexpression decreased OTA-induced oxidative stress in PK15 cells

**Figure 7:**
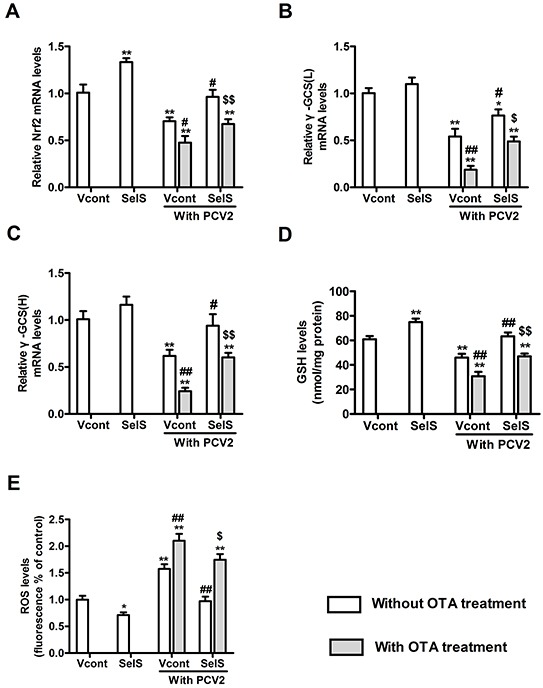
SelS overexpression decreased OTA-induced oxidative stress in PK15 cells PK15 cells overexpressing vector or SelS were cultured for 12h and then incubated for an additional 60 h with PCV2 in the presence or absence of 0.05 μg/ml OTA. Cellular levels of Nrf2 **A.** and γ-GCS **B, C.** mRNA, GSH **D.** and ROS **E.** were assayed as described in the Materials and Methods. Data are presented as means ± SE of three independent experiments. *P < 0.05 and **P < 0.01 vs. control (without OTA and PCV2). Within the PCV2 infection group, *^#^P* < 0.05 and ^##^*P* < 0.01 vs. Vcont cells without OTA treatment and ^**$**^*P* < 0.05 and ^**$$**^*P* < 0.01 vs. Vcont cells with OTA treatment.

To investigate the mechanism by which SelS overexpression blocked OTA-induced promotion of PCV2 replication, levels of Nrf2 mRNA, γ-GCS mRNA, GSH, and ROS were determined. Cells were cultured at a density of 1.5 × 10^5^/well in 6-well plates for 12h and then incubated with or without PCV2 in the presence or absence of 0.05 μg/ml OTA for an additional 60 h. As shown in Figure 7, in cells without OTA and PCV2 treatment, SelS overexpression increased Nrf2 mRNA and GSH levels (Figure 7A, 7D) and decreased ROS levels (Figure 7E). SelS overexpression had no effect on γ-GCS mRNA levels (Figure 7B, 7C). PCV2 infection decreased Nrf2 mRNA levels (Figure 7A), γ-GCS (Figure 7B, 7C) mRNA levels and reduced GSH levels (Figure 7D), and increased ROS levels (Figure 7E) compared to the vector control group without PCV2 infection, and SelS overexpression reversed these changes. OTA enhanced the PCV2-induced decreases in Nrf2 mRNA (Figure 7A), γ-GCS mRNA (Figure 7B, 7C), and GSH levels (Figure 7D), as well as the increase in ROS levels (Figure 7E). SelS overexpression reversed these OTA-induced changes compared to the vector groups (Figure 7) (*P* < 0.05). These results suggest that SelS overexpression may block OTA-induced PCV2 replication promotion by inhibiting oxidative stress.

### SelS overexpression inhibits OTA-induced p38 phosphorylation in PK15 cells

**Figure 8:**
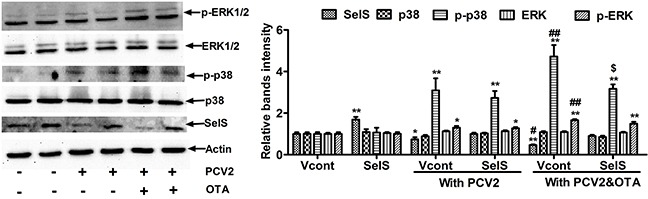
SelS overexpression inhibits OTA-induced p38 phosphorylation in PK15 cells PK15 cells overexpressing vector or SelS were cultured for 12 h and then incubated for an additional 60 h without or with PCV2 in the presence or absence of 0.05 μg/ml OTA. Cells were assayed for p38 phosphorylation as described in the Materials and Methods. Data are presented as means ± SE of three independent experiments. *P < 0.05 and **P < 0.01 vs. Vcont (without OTA and PCV2). Within the PCV2 infection group, *^#^P* < 0.05 and ^##^*P* < 0.01 vs. Vcont cells without OTA treatment and ^**$**^*P* < 0.05 and ^**$$**^*P* < 0.01 vs. Vcont cells with OTA treatment.

Next, we investigated whether SelS overexpression blocked OTA-induced promotion of PCV2 replication via the p38 and ERK1/2 MAPK signaling pathways. Cells were cultured at a density of 2 × 10^5^/well in 6-well plates for 12h and then incubated with or without PCV2 in the presence or absence of 0.05 μg/ml OTA for an additional 60 h. As shown in Figure 8, in cells without OTA and PCV2 treatment, SelS overexpression increased SelS protein levels, but had no effect on p38, p-p38, ERK1/2, or p-ERK1/2 protein levels. PCV2 infection alone increased p38 and ERK1/2 phosphorylation. OTA treatment further increased p38 and ERK1/2 phosphorylation levels in PCV2-infected cells, and SelS overexpression blocked OTA-induced p38, but not ERK1/2, phosphorylation. These results suggest that SelS overexpression may block OTA-induced promotion of PCV2 replication through the p38 MAPK signaling pathway, and not the ERK1/2 pathway.

### Both oxidative stress and p38 mediate the ability of SelS overexpression to block OTA-promoted PCV2 replication in PK15 cells

**Figure 9:**
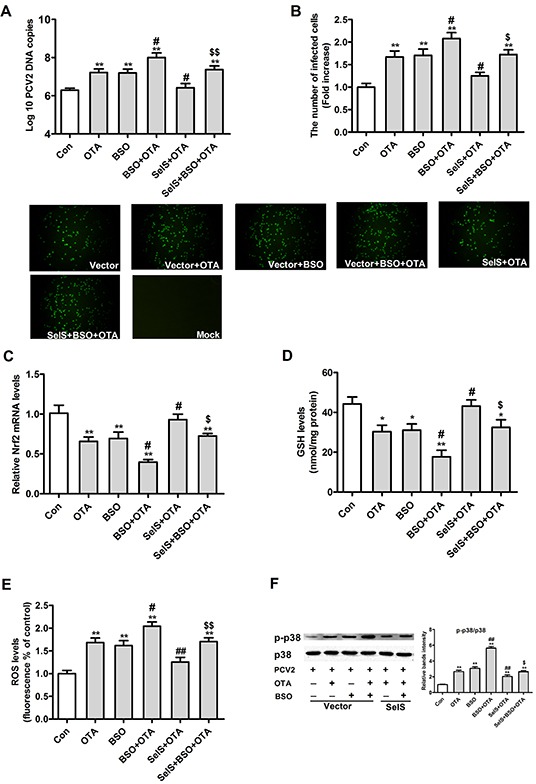
Effects of SelS overexpression and/or OTA and/or BSO on PCV2 replication, oxidative stress and p38 phosphorylation in PCV2-infected PK15 cells PK15 cells overexpressing vector or SelS were cultured for 12 h and then incubated for an additional 60 h with PCV2 in the presence or absence of 0.05 μg/ml OTA and/or 50 μM BSO. Cells were harvested and assayed for PCV2 DNA copies **A.** the number of infected cells **B.** and levels of Nrf2 **C.** GSH **D.** ROS **E.** and p38 phosphorylation **F.** as described in the Materials and Methods. Data are presented as means ± SE of three independent experiments. *P < 0.05 and **P < 0.01 vs. control. ^#^*P* < 0.05 and ^##^*P* < 0.01 vs. OTA treatment. ^$^*P* < 0.05 and ^$$^*P* < 0.01 vs. OTA and SelS.

Because oxidative stress can activate the p38 MAPK signaling pathway [32, 33], we investigated whether SelS overexpression in PK15 cells blocked OTA-induced promotion of PCV2 replication by activating p38 MAPK in response to oxidative stress. To address this question, we assessed the effects of 50 μM buthionine sulfoximine (BSO) on PCV2 replication, oxidative stress, and p38 phosphorylation. As shown in Figure 9, BSO increased numbers of PCV2 DNA copies (Figure 9A) and infected cells (Figure 9B), and reversed the blocking effect of SelS overexpression on OTA-induced promotion of PCV2 replication. SelS overexpression blocked OTA-induced decreases in Nrf2 mRNA (Figure 9C) and GSH levels (Figure 9D) and increases in ROS (Figure 9E) levels and p38 phosphorylation PCV2-infected PK15 cells. BSO reversed these changes, but did not change the effects of SelS overexpression on total p38 levels (Figure 9F). These results indicate that SelS overexpression may block OTA-induced promotion of PCV2 replication by inhibiting oxidative oxidative stress and p38 phosphorylation.

### SelS gene expression after transfection of SelS-siRNA into PK15 cells

**Figure 10:**
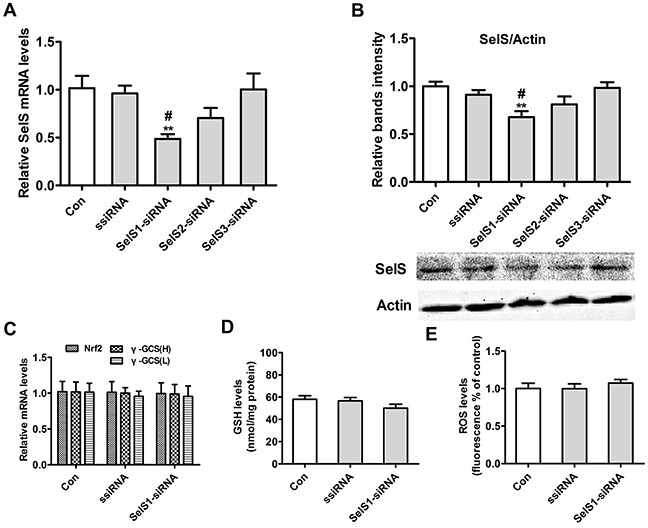
SelS gene expression after transfection of SelS-siRNA into PK15 cells Vector-expressing PK15 cells were incubated with or without SelS-siRNA. Cell samples were assayed for levels of SelS mRNA **A.** actin and SelS protein **B.** Nrf2 and γ-GCS mRNA **C.** GSH **D.** and ROS **E.** Data are presented as means ± SE of three independent experiments. *P < 0.05 and **P < 0.01 vs. control. *^#^P* < 0.05 and ^##^*P* < 0.01 vs. ssiRNA.

To select the SelS-siRNA that interfered most with SelS expression, we transiently transfected three pig SelS-siRNAs into Vector-PK15 cells and measured SelS mRNA and protein levels. As shown in Figure 10, transfection of SelS1-siRNA decreased SelS mRNA (Figure 10A) and protein levels (Figure 10B). However, transfection of SelS2-siRNA and SelS3-siRNA did not decrease SelS expression (Figure 10B). Transfection of Vector-PK15 cells with SelS1-siRNA had no effect on Nrf2 mRNA, γ-GCS mRNA (Figure 10C), GSH (Figure 10D), or ROS levels (Figure 10E). Because SelS1-siRNA effectively knocked down SelS expression, it was used in subsequent experiments.

### SelS knockdown enhanced OTA-induced oxidative stress and p38 phosphorylation in PCV2-infected PK15 cells

**Figure 11:**
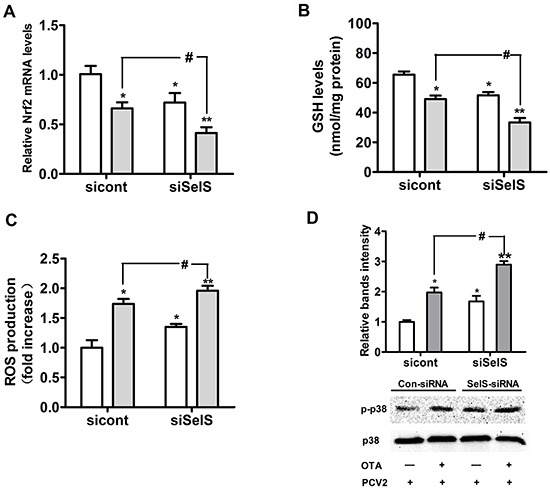
SelS knockdown enhanced OTA-induced oxidative stress and p38 phosphorylation Vector-expressing PK15 cells were cultured for 12 h and then incubated for an additional 60 h with PCV2 in the presence or absence of 0.05 μg/ml OTA. Cells were assayed for levels of Nrf2 mRNA **A.** GSH **B.** ROS **C.** and p38 phosphorylation **D.** Data are presented as means ± SE of three independent experiments. *P < 0.05 and **P < 0.01 vs. sicont without OTA treatment. ^#^*P* < 0.05 and ^##^*P* < 0.01 vs. sicont with OTA treatment.

To confirm that SelS overexpression inhibits oxidative stress, we used SelS-specific siRNA to knock down SelS expression in Vector-PK15 cells. Vector-PK15 cells were cultured overnight and then transfected with control-siRNA or SelS-siRNA. After 5 h of transfection treatment, the medium was removed and fresh basal medium was added, and cells were then incubated with PCV2 in the presence or absence of 0.05 μg/ml OTA for an additional 60 h. As shown in Figure 11, 0.05 μg/ml OTA decreased Nrf2 mRNA (Figure 11A) and GSH levels (Figure 11B) and increased ROS levels (Figure 11C) and p38 phosphorylation (Figure 11D) in PCV2-infected cells. SelS knockdown decreased Nrf2 mRNA (Figure 11A) and GSH levels (Figure 11B) and increased ROS levels (Figure 11C) and p38 phosphorylation (Figure 11D) compared to control cells with or without OTA treatment (Figure 11). These results suggest that SelS knockdown enhances OTA-induced oxidative stress and p38 phosphorylation.

### SelS knockdown promoted OTA-induced PCV2 replication in PK15 cells

**Figure 12:**
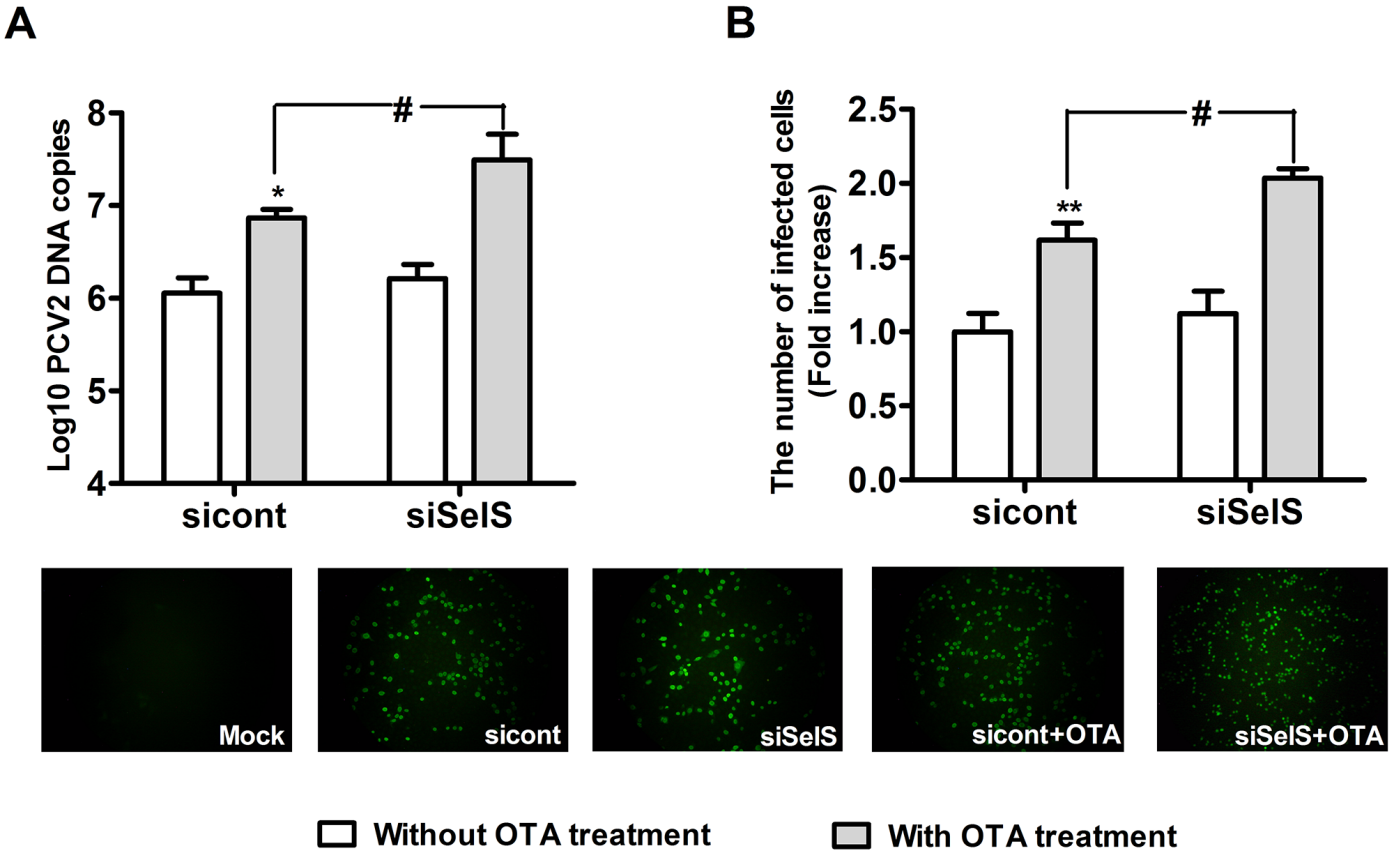
SelS knockdown promoted OTA-induced PCV2 replication Vector-expressing PK15 cells were cultured for 12 h and then incubated for an additional 60 h with PCV2 in the presence or absence of 0.05 μg/ml OTA. Cells were assayed for PCV2 viral DNA copies **A.** using real-time PCR and the number of infected cells **B.** using IFA. Data are presented as means ± SE of three independent experiments. *p < 0.05 and **p < 0.01 sicont without OTA treatment. *^#^p* < 0.05 and ^##^ *p* < 0.01 sicont with OTA treatment.

Next, we used SelS-siRNA to confirm that SelS overexpression inhibits OTA-induced promotion of PCV2 replication. Vector-PK15 cells were cultured overnight and then transfected with a control-siRNA or SelS-siRNA. After 5 h of transfection treatment, the medium was removed and fresh basal medium was added, and cells were then incubated with PCV2 in the presence or absence of 0.05 μg/ml OTA for an additional 60 h. As shown in Figure 12, a trend towards increased PCV2 replication was observed in SelS-knockdown cells without OTA treatment compared to the control cells (Figure 12A, 12B), but this effect was not significant (*P* > 0.05). OTA at 0.05 μg/ml increased the numbers of PCV2 DNA copies and infected cells (Figure 12A, 12B). SelS knockdown enhanced the OTA-induced promotion of PCV2 replication compared to the control-siRNA group (Figure 12A, 12B) (*P* > 0.05). These results suggest that SelS knockdown promotes OTA-induced PCV2 replication in PK15 cells.

## DISCUSSION

Se is an essential trace element that exerts biological effects via incorporation into selenoproteins [34, 35], which have selenocysteine (Sec) as the 21st amino acid residue [19]. The most remarkable trait of selenoprotein biosynthesis is the co-translational insertion of Sec by the recoding of a naturally occurring UGA stop codon [20, 21]. The Sec Insertion Sequence (SECIS) element located in the 3′-UTR of all selenoprotein mRNA is required for incorporation of Sec into the nascent selenoprotein polypeptides in response to the UGA codon [22, 23]. In addition to the SECIS element, the three proteins selenocysteyl-tRNA (Sec-tRNA^Sec^), SECIS binding protein-2 (SBP2), and a dedicated eukaryotic elongation factor (eEFSec) are needed for successful conversion of the UGA codon into a Sec residue [36-38]. Because UGA recoding by the Sec machinery is believed to be very inefficient owing to RF2-mediated termination at UGA, cloning selenoproteins is relatively difficult.

Approximately twenty-five known selenoproteins have been characterized [39], and they are known for their roles in catalyzing redox reactions and defending cells against oxidative stress [34]. Among the known selenoproteins, SelS is particularly important, and many molecular techniques, including overexpression and knockdown in human cell lines, have been developed to study its functions [24, 30]. Previous studies indicate that SelS has antioxidant functions in humans [29, 30]. However, the functions of SelS and effects of overexpression in pigs have not been studied. Here, we successfully constructed a pig SelS-plasmid and PK15 cell lines overexpressing SelS for the first time. SelS mRNA and protein levels in PK15 cells with SelS overexpression were about two times higher than those in control cells, similar to results observed in humans [30].

SelS overexpression also reverses H_2_O_2_-induced decreases in cell viability and SOD activity and increases in MDA, and SelS knockdown enhances these H_2_O_2_-induced changes [30]. However, the effects of SelS on oxidative stress in pigs have not been examined. In the present study, cell viability was not affected by transfection of pc-SelS into PK15 cells. However, SelS overexpression increased Nrf2 mRNA and GSH levels and decreased ROS levels in PK15 cells. These results suggest that pig SelS also has antioxidation.

It has previously been reported that some selenoproteins may regulate viral infections. For example, GPx1 knockdown increased rates of poliovirus and PCV2 infection [13, 15], TR1-siRNA increased HIV-1 replication and Tat-dependent transcription in human macrophages [40], and high GPx2 expression decreased hepatitis C virus RNA levels in human liver [41]. However, little is known concerning the relationship between SelS and PCV2 replication. Here, we demonstrated that SelS overexpression blocks OTA-induced promotion of PCV2 replication. PCV2 infection decreased SelS expression, and OTA treatment enhanced this decrease. Furthermore, Se supplementation and SelS overexpression blocked, and siRNA-induced SelS knockdown enhanced, the OTA-induced promotion of PCV2 replication. Other selenoproteins, especially GPx1, may have similar antiviral effects; we previously demonstrated that GPx1 knockdown enhanced the H_2_O_2_-induced promotion of PCV2 replication [15]. We will examine the relationship between other selenoproteins with antioxidant effects and PCV2 replication in future studies.

Our previous study showed that OTA-induced oxidative stress promoted PCV2 replication [5]. In addition, it has been reported that SelS has antioxidation [30, 42], and the present work indicates that SelS overexpression increases antioxidant activity in PK15 cells. We speculate that SelS overexpression blocks OTA-induced promotion of PCV2 replication by reducing oxidative stress. In the present study, SelS overexpression reversed OTA-induced decreases in Nrf2 mRNA, γ-GCS mRNA, and GSH levels, as well as OTA-induced increases in ROS levels in PCV2-infected PK15 cells. SelS knockdown had the opposite effects on the above OTA-induced changes.

BSO, a specific inhibitor of glutamate-cysteine ligase which causes oxidative stress [43, 44] and promotes PCV2 replication [4], was used in the present work to confirm the role of SelS. BSO decreased Nrf2 mRNA, γ-GCS mRNA, and GSH levels, increased ROS production, and eliminated the blocking effects of SelS overexpression on OTA-promoted PCV2 replication, consistent with previous work [4, 43]. These results strongly support the hypothesis that SelS overexpression blocks OTA-induced promotion of PCV2 replication by inhibiting oxidative stress.

Several previous studies indicate that Se regulates MAPK signaling pathways. Se supplementation suppresses LPS-induced ERK, JNK, and p38 phosphorylation [45]. In contrast, Se deficiency upregulates p38, p-p38, p-JNK, and p-ERK protein expression in chickens [46]. GPx1 knockdown also increases p38 phosphorylation in mice [47]. In addition, PCV2 activates the p38, ERK, or JNK signaling pathways to promote its replication [48, 49], and our previous work indicated that OTA promotes PCV2 replication by activating the p38 and ERK signaling pathways [5]. Thus, we propose that the blocking effects SelS overexpression are due in part to inhibition of the p38 and ERK signaling pathways.

The present results show that 0.05 μg/ml OTA induced p38 and ERK1/2 phosphorylation. SelS overexpression in turn inhibited OTA-induced p38 phosphorylation in PCV2-infected cells, but had no effect on ERK1/2 phosphorylation. In contrast, SelS knockdown enhanced OTA-induced p38 phosphorylation. Because SelS overexpression increased Nrf2 mRNA and GSH levels, depleted ROS production, and inhibited p38 phosphorylation, we hypothesize that SelS overexpression blocks OTA-induced promotion of PCV2 replication by inhibiting oxidative stress-induced p38 phosphorylation. Indeed, BSO enhanced OTA-induced p38 phosphorylation and reversed the inhibition of p38 phosphorylation that resulted from SelS overexpression. However, SelS overexpression does not seem to inhibit oxidative stress by acting on the ERK 1/2 pathway.

In conclusion, we successfully constructed a pCDNA3.1-SelS plasmid and PK15 cell lines that overexpress SelS. SelS overexpression blocked OTA-induced promotion of PCV2 replication, and SelS knockdown had the opposite effect. In addition, this blocking effect of SelS was likely due to its ability to inhibit oxidative stress-induced p38 signaling pathway activation. Thus, our work provides new insights regarding the relationship between SelS and viral infection and describes an antiviral mechanism of action for Se.

## MATERIALS AND METHODS

### Cell culture and virus infection

PK15 cells free of PCV were provided by the China Institute of Veterinary Drug Control. The cells were maintained in Dulbecco's minimal Eagle's medium (DMEM, Invitrogen, USA) supplemented with heat-inactivated 8% fetal bovine serum (FBS), penicillin (100 U/ml), and streptomycin (100 μg/ml) at 37°C in a humidified atmosphere containing 5% CO_2_. PCV type was determined through sequencing (Invitrogen). PCV2 stocks were generated using the following procedure: PK15 cells were infected with PCV2 at a multiplicity of infection (MOI) of 1 when they had reached approximately 40%–50% confluence. After 1 h absorption, the inoculum was removed, and the cell monolayer was washed three times with phosphate-buffered saline (PBS). DMEM medium including 2% FBS, penicillin (100 U/ml), and streptomycin (100 μg/ml) was subsequently added, and incubation was continued at 37°C for 72 h. Next, the infected cells were subcultured in DMEM and serial passage was performed to isolate PCV2 from PK15 cells. The virus harvested at each passage was stored at −80°C.

### Construction of the SelS over-expression plasmid (pc-SelS)

A specific primer for the analysis of SelS was designed using Primer Premier Software (PREMIER Biosoft International, Palo Alto, CA, USA) according to the nucleotide sequence of porcine SelS [GeneBank: AY609646.1]. Restriction enzyme sites were introduced into the forward primer (XhoI at the 5′ end) and the reverse primer (EcoRI at the 3′ end) to enable subcloning of the amplified fragment. The forward primer (5′-GCGAATTCATGGAGCAGGACGGGGACC-3′) and the reverse primer (5′-GGCTCGAGCAGAAACAACCCTATCAAC-3′) were used to amplify a 1029-bp fragment of the SelS gene from porcine kidney tissue. Total RNA was extracted from porcine kidney tissue using the RNAiso Plus kit (TaKaRa, China) according to the manufacturer's protocol. Potential DNA contamination of the extraction was eliminated using the DNA-Free kit (TaKaRa) and RNA quality was assessed by the absorbance ratio at 260/280 nm. First-strand cDNA was synthesized and PCR was carried out using the ABI Prism (Applied Biosystems, USA). The PCR product was cloned into the pMD19-T Simple Vector and then digested with XhoI and EcoRI. The digested SelS DNA fragment was purified and subcloned into the pcDNA3.1 eukaryotic expression vector to generate the pcDNA3.1-SelS recombinant plasmid (pc-SelS) and sequenced.

### Construction of the PK 15 cell lines with over-expression of SelS

pc-SelS was transfected using X-tremeGENE transfection reagent (Roche) into PK15 cells cultured in Dulbecco's modified Eagle's medium (DMEM, Invitrogen) supplemented with 8% FBS. To select stable transfectants, cells were grown in complete medium supplemented with 400 mg/ml Geneticin G418 antibiotics (Invitrogen). Control cells were prepared by transfecting PK15 cells with the empty pCDNA3.1 construct and then selecting resistant clones as above. Positive and stably transfected PK15 cells in DMEM with 8% FBS were analyzed for porcine SelS mRNA levels by real-time PCR and for SelS protein expression by western-blot.

### Cell viability assay

PK15 cells were cultured for 72 h in 96-well plates and subjected to the colorimetric 3-(4,5-Dimethylthiazol-2-yl)-2,5-diphenyltetrazoliumbromide (MTT) assay (Sigma, USA). Absorbance was measured at 490 nm with a secondary wavelength of 650 nm. All tests were performed with four replicates. Cell viability was calculated as % of control cells.

### SYBR green real-time PCR

SYBR green real-time PCR was performed to determine the levels of SelS, Nrf2 mRNA, and γ-GCS mRNA and the number of PCV2 DNA copies in PK15 cells. For mRNA measurements, primers for analysis of SelS were designed using Primer Premier Software (PREMIER Biosoft International, Palo Alto, CA, USA) based on known porcine sequences. The forward primer (5′-GGAAGCGTCAGGAAGAAG-3′) and the reverse primer (5′-TTAGCCTCATCCACCAGAT-3′) were used to amplify a 176-bp fragment of the SelS gene. The primer sequences for β-actin (a control reference gene), Nrf2, and γ-GCS were obtained from a published article [5]. Total RNA was extracted from PK15 cells using the RNAiso Plus kit (TaKaRa, China) according to the manufacturer's protocol. Potential DNA contamination of the extraction was eliminated using the DNA-Free kit (TaKaRa) and RNA quality was assessed by the absorbance ratio at 260/280 nm. First-strand cDNA was synthesized and PCR was carried out using the ABI Prism Step One Plus detection system (Applied Biosystems, USA) as described previously [50]. The relative mRNA levels of target genes were determined using the Δ cycle threshold (ΔCt) method with β-actin serving as a reference gene.

For PCV2 measurements, DNA was extracted using the TaKaRa DNA Mini kit (TaKaRa, China) and the purified DNA was used as the template for PCR amplification. SYBR green real-time PCR was carried out using the ABI Prism Step One Plus detection system (Applied Biosystems, USA). A recombinant pMD19 plasmid vector (TaKaRa) containing a PCV2 genome insert as a reference and a TaKaRa SYBR green real-time PCR kit (TaKaRa, China) were used.

### Determination of intracellular ROS levels

Intracellular ROS levels in PK15 cells were measured with MitoSOX Red mitochondrial superoxide indicator (Invitrogen, USA) as described previously [51]. Briefly, after removing the culture medium, cells were washed three times with PBS. MitoSOX Red mitochondrial superoxide indicator, diluted to a final concentration of 4 μM with serum-free DMEM, was added to the cells and incubated for 10 min at 37°C, while protecting from light. Cells were then washed three times with PBS. The cells were re-suspended in PBS and fluorescence was measured immediately by FACS Calibur flow cytometer. Intracellular ROS levels as indicated by fluorescence intensity were calculated as a percentage of control cell fluorescence.

### Determination of intracellular GSH levels

Cell extracts were prepared by sonication (SonicsVCX105, USA) in ice-cold PBS and centrifuged at 12,000 rpm for 20 min to remove debris. The supernatant fluid was collected and GSH levels determined spectrophotometrically at 412 nm by reaction with 5, 5′-dithiobis (2-nitrobenzoicacid) as described previously [15] using commercially available kits (Jiancheng, China). Total protein concentration was determined using a BCA protein assay kit (Beyotime, China). The data are expressed as nanomoles of GSH per milligram of protein.

### Indirect immunofluorescence assay (IFA)

PCV2-infected cells were identified by IFA as described previously [5]. PK15 cells were washed with PBS containing 0.1% Tween 20 (PBST) and fixed in 4% paraformaldehyde. After three washes, the cells were perforated with 0.1% Triton X-100 and then blocked in PBST containing 1% bovine serum albumin (BSA) at 37°C for 45 min to prevent nonspecific binding. Next, the cells were incubated at 37°C for 1 h with porcine anti-PCV2 antibody (UnivBiotech, China) diluted in PBST containing 1% BSA (PBSTB) (1:50), and after three washes with PBST, FITC-conjugated rabbit anti-pig antibody (Sigma; diluted 1:100 in PBSTB) was added and incubated for 1 h at 37°C. After three washes, the cells were examined under a fluorescence microscope. Cells positive for PCV2 viral antigens were counted in six fields of view.

### Western blotting

Cells were collected in 80 μl lysis buffer containing protease inhibitors (Beyotime, China) and were sonicated (SonicsVCX105, USA). The lysate was centrifuged at 12,000 rpm for 20 min at 4°C and the supernatant was immediately collected for use. Protein concentration was determined using the BCA kit (Beyotime, China). Fifty μg of protein were diluted in sample loading buffer and heated at 95°C for 5 min. The denatured proteins were resolved by 12% sodium dodecyl sulphate-polyacrylamide gel electrophoresis (SDS–PAGE), and transferred to polyvinylidene difluoride (PVDF) membranes. The membranes were incubated for 2 h at RT in Tris-buffered saline (TBS) containing 5% milk (for SelS) or BSA (for β-actin, p38, p-p38, ERK1/2, and p-ERK1/2), and 0.1% Tween 20 (TBST), followed by overnight incubation at 4°C in specific primary antibodies (anti-SelS from Santa Cruz Biotechnology, diluted 1/500; anti-β-actin, anti-p38, anti-p-p38 anti-ERK1/2, and anti-p-ERK1/2 from Cell Signaling, diluted 1/1000). The membranes were washed and incubated in HRP-conjugated secondary antibody (polyclonal anti-rabbit–horseradish peroxidase from Sigma) at RT for 1h. The blots were visualized and analyzed by a Luminescent Image Analyzer (FUJIFILM LAS-4000) and expressed as a percentage respect to the control group.

### Small interfering RNA (siRNA) transfection

Three SelS-specific siRNAs were designed using the sequence of *Sus scrofa* SelS mRNA (GenBank Accession No. NM_001164113) and Invitrogen BlockiT RNAi designer). Control siRNA sequences were obtained from a published paper [5]. The SelS-specific siRNA sequences were 5′-GCUUUAGCAGCAGCUCGUUtt-3′, 5′-GAAGCUAAGACAGCUCGAAtt-3′, and 5′-GCUAA GACAGCUCGAAGAAtt-3′. The three double-stranded RNAs were synthesized by Invitrogen. Duplexes were re-suspended in RNA-free water to obtain 20 μM solutions before use. The duplexes were transiently transfected into PK15 cells via liposomes using X-tremeGENE transfection reagent (Roche). Briefly, PK15 cells in DMEM with 8% FBS without antibiotics were cultured overnight at 37°C. When cells were 30–50% confluent, siRNA was introduced using the X-tremeGene siRNA transfection reagent according to the manufacturer's protocol. Transfection reagent and siRNA (5:1) were added to each well and incubated for 5h. The cells were then washed with DMEM and transferred to DMEM with 4% FBS. To determine the interference efficiency of SelS-siRNA, porcine SelS mRNA expression was analyzed by real-time PCR and protein levels by western-blot. The SelS-siRNA with the most efficient interference was selected for use in the present experiment.

### Statistical analysis

One-way analysis of variance (ANOVA) followed by Duncan's multiple range tests were used to determine differences between means using the SPSS computer program for Windows (version 17.0). Results are expressed as the mean ± standard error (SE). *P*-values of less than 0.05 were considered statistically significant.
